# In Silico Optimization of Charge Separating Dyes for Solar Energy Conversion

**DOI:** 10.1002/cssc.202200594

**Published:** 2022-06-22

**Authors:** Jan Paul Menzel, Yorrick Boeije, Tijmen M. A. Bakker, Jelena Belić, Joost N. H. Reek, Huub J. M. de Groot, Lucas Visscher, Francesco Buda

**Affiliations:** ^1^ Leiden Institute of Chemistry Leiden University PO Box 9502 2300 RA Leiden Netherlands; ^2^ Van't Hoff Institute for Molecular Sciences University of Amsterdam 1098XH Amsterdam Netherlands; ^3^ Department of Chemistry and Pharmaceutical Sciences Vrije Universiteit Amsterdam 1081 HV Amsterdam Netherlands

**Keywords:** charge transfer, computational chemistry, dye-sensitization, photoelectrochemistry, quantum propagation

## Abstract

Dye‐sensitized photoelectrochemical cells are promising devices in solar energy conversion. However, several limitations still have to be addressed, such as the major loss pathway through charge recombination at the dye‐semiconductor interface. Charge separating dyes constructed as push‐pull systems can increase the spatial separation of electron and hole, decreasing the recombination rate. Here, a family of dyes, consisting of polyphenylamine donors, fluorene bridges, and perylene monoimide acceptors, was investigated in silico using a combination of semi‐empirical nuclear dynamics and a quantum propagation of photoexcited electron and hole. To optimize the charge separation, several molecular design strategies were investigated, including modifying the donor molecule, increasing the π‐bridge length, and decoupling the molecular components through steric effects. The combination of a triphenylamine donor, using an extended 2‐fluorene π‐bridge, and decoupling the different components by steric hindrance from side groups resulted in a dye with significantly improved charge separation properties in comparison to the original supramolecular complex.

## Graphical abstract

**A charged situation**: Charge‐separating donor‐π‐acceptor dyes can lower the recombination losses in dye‐sensitized solar and photoelectrochemical cells. Here, charge separators based on polyphenylamine donors, fluorene bridges, and perylene monoimide acceptors are optimized in silico using a quantum‐classical approach combining nuclear and electronic dynamics. Based on the findings, a charge separator is proposed with an increased charge accumulation by approximately 50 %.

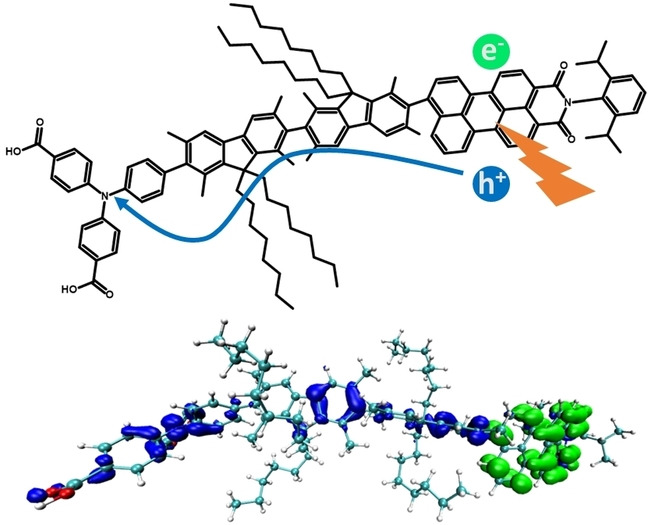

## Introduction

Dye‐sensitized photoelectrochemical cells (DS‐PECs) have shown great promise for the production of renewable fuels, where semiconductor electrodes are sensitized with chromophores that absorb light in the visible range.^[1]^ On the photoanode side, the photoexcited dyes inject the excited electrons into the semiconductor electrode to be used on the photocathode for fuel production. The hole left at the dye can then be picked up by a water oxidation catalyst (WOC) to split water into molecular oxygen and protons, regenerating the ground state of the molecular chromophore.^[2]^

**Scheme 1:**
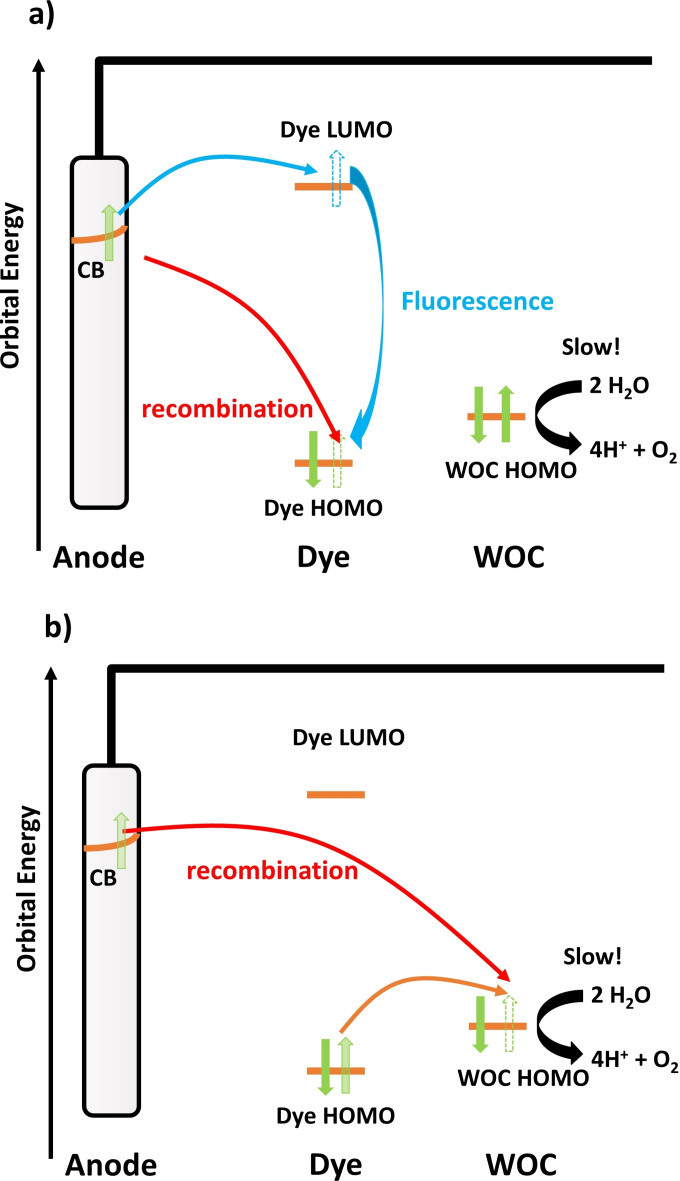
Energy loss pathways in a dye‐sensitized photoanode. (a) After electron injection from the dye LUMO into the conduction band (CB) of the anode, the hole on the dye HOMO can be either filled by direct recombination (red) with the photoexcited electron from the anode or by fluorescence after back transfer of the electron from the anode (blue). (b) Following the hole transfer to the WOC, recombination of the photoexcited electron from the anode (red) or back transfer to the dye's HOMO (orange), which is followed by the processes in (a) can lead to the loss of the excited‐state energy.

Water oxidation, however, is a slow process involving storage of intermediates on long time scales in comparison to the time scale of an electron injection from the photoexcited dye into the semiconductor anode.^[3]^ Due to the slow consumption of holes in the chemical conversion of water to molecular oxygen and protons, the holes can be refilled as shown in Scheme 1. This process may occur through a direct recombination of the photoexcited electron in the anode and the hole as seen in Scheme 1a (red arrow), leading to a full deactivation and energetic losses through heat. Another loss pathway shown in blue is through back transfer of the excited electron to the dye followed by fluorescence, releasing the excess energy in the form of a photon.

Deactivation is also possible after initially successful charge separation and hole transfer to the WOC as shown in Scheme 1b. This involves either direct recombination with the photoexcited electron from the semiconductor (red arrow), or back transfer of the hole to the dye as shown in orange, opening up the loss pathways given in Scheme 1a. All these processes lead to the loss of the absorbed photon energy. The factor determining the losses is the ratio between the rates of the competing processes. Furthermore, since four redox equivalents need to be transferred to the WOC for each oxygen molecule released, involving several oxidative states with different potentials, finding appropriate dyes with tuned redox properties that can oxidize each respective state also leads to challenges in fine tuning of the electronic states and reaction rates.

The creation of the charge‐separated state through the electron injection process into TiO_2_ is extremely fast, often in the sub‐picosecond or picosecond time frame.^[4]^ On the contrary, the rate of the slowest oxidation step of typical WOCs is in the order of micro‐ to milliseconds depending on the concentration of the oxidant (e. g., compound 1 of ref. [5]: 2.3×10^5^ m ^−1^ s^−1^ at 35 °C with Ce^IV^). The time mismatch between these two processes is therefore several orders of magnitude, over which the charge separated state needs to be stabilized. While the last decade has seen important improvements in the turnover frequency of WOCs,[^5^, ^6^] reducing the charge recombination and back transfer rates is an important factor to minimize losses.

**Scheme 2:**
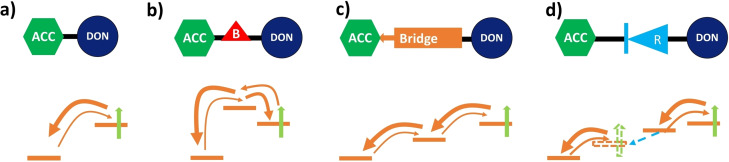
Schematic representation of different approaches to reduce the back transfer from the acceptor (ACC) towards the donor (DON) after initial charge separation. (a) Direct attachment of acceptor and donor allows for relatively easy back transfer. (b) Introducing an energetic barrier reduces the rate of initial charge transfer, but also reduces the back transfer rate significantly. (c) Spatial separation via a bridge leads to lower back transfer probability. (d) Introduction of a molecular rectifier, which through asymmetrical HOMO and LUMO localization or resonant conformational changes increases charge flow in one direction, while decreasing it in the opposite.

In general, several strategies can be applied to reduce the probability for back transfer of an electron or hole in a donor‐acceptor system. In Scheme 2, some of these possible approaches are shown schematically. When attaching the electron acceptor (e. g., the dye) directly to the donor, as shown in Scheme 2a, back transfer, though slower than forward charge transfer, can occur relatively unhindered. One way to reduce the rate of back transfer is to introduce a barrier between donor and acceptor by including a molecular system with a highest occupied molecular orbital (HOMO) energy higher than that of both donor and acceptor as seen in Scheme 2b.

While the initial transfer rate is decreased, since this barrier needs to be overtaken, the back transfer rate is even more reduced. Another possible strategy, for example used in natural photosynthesis,^[7]^ is separating electron and hole spatially as quickly as possible by introducing a bridge consisting of one or more electronic systems with clear energy gradients of the involved orbitals as shown in Scheme 2c. Finally, molecules where HOMO and lowest unoccupied molecular orbital (LUMO) are on spatially different components, or molecules that change conformation upon population of the LUMO (blue arrow), are efficient ways to allow for charge transfer in one direction but inhibit migration in the opposite direction as shown schematically in Scheme 2d. These molecules are called molecular rectifiers.^[8]^

A strategy combining some of the approaches described above for reducing charge recombination is the introduction of charge‐separating dyes commonly used on the other half‐cell reaction, the photo‐driven hydrogen evolution on the photocathode.^[9]^ They consist of π‐conjugated donor‐acceptor molecules, with a donor component that accumulates hole density, an acceptor that accumulates electron density, and a π‐system bridging the two. This arrangement of donor‐π system‐acceptor (DON‐π‐ACC) is often also called a push‐pull system, named after their respective tendencies of pushing (donor) or pulling (acceptor) electron density.^[10]^ The electronic properties of these systems are designed to allow for efficient intramolecular charge transfer. The explicit molecular systems can vary quite significantly, depending on the requirements on the molecules in their device implementation. Within the context of dye‐sensitized solar cells (DSSCs) and DS‐PECs, some common donor molecules include diphenylamines (DPA)[^9a^, ^9b^, ^10a^, ^11^] and triphenylamines (TPA)[^1b^, ^9c^, ^12^] as well as their derivatives.^[13]^ The π‐conjugated molecular spacer or bridge is often an extended system, such as thiophene derivatives[^9c^, ^12b^, ^12e^, ^12f^, ^12g^, ^13a^, ^14^] or fluorenes.[^9a^, ^9b^, ^10a^, ^13a^] The bridge can, however, also involve a photosensitizer such as porphyrin[^12c^, ^13c^, ^15^] that donates an electron to the acceptor while the hole is transferred to the donor. If either the bridge or the donor is the photosensitizer, the acceptor can be non‐photoactive, such as benzoic acids.[^12b^, ^12c^, ^13c^, ^15^] Cyano acrylic acids[^11^, ^13a^, ^14^] are also used as acceptors, but various fragments can be chosen to tune the desired energy levels.^[12e]^ A common photoactive acceptor is perylene‐monoimide (PMI).[^9^, ^12f^, ^12g^] The dyes are often equipped with alkyl or alkoxy chains to act as a protective layer for the respective electrode and anchoring groups, preventing electrolyte interference, but also dye aggregation.[^9^, ^12b^, ^13a^, ^13c^, ^15^]

Implemented within a larger donor‐acceptor system, these dyes can act as barrier, bridge, or molecular rectifier, for either electron or hole, allowing for easy transfer in one direction while inhibiting back transfer. Organic molecules with these charge‐separating qualities are therefore of interest in both half cells of DS‐PECs whenever charge recombination becomes a major loss pathway.

Here, we do an in silico investigation and optimization of such push‐pull systems for efficient charge separation and back transfer suppression using a quantum‐classical semi‐empirical approach: Tight‐binding GFN‐xTB^[16]^‐based nuclear classical dynamics in the ground state are followed by a wave packet quantum propagation of photoexcited electron and hole on these nuclear trajectories using an extended Hückel Hamiltonian.^[17]^ Including nuclear dynamics in the description of photoinduced charge transfer is crucial since nuclear motion has been shown to influence electron transfer processes significantly.[^4b^, ^18^] GFN‐xTB has been optimized for a proper description of molecular geometries and frequencies and has proven reliable in a wide variety of systems.^[19]^ The quantum propagation method has also been applied to describe the photoinduced electron injection in dye‐sensitized photoanodes.[^3^, ^4b^, ^18a^, ^20^]

**Figure 1:**
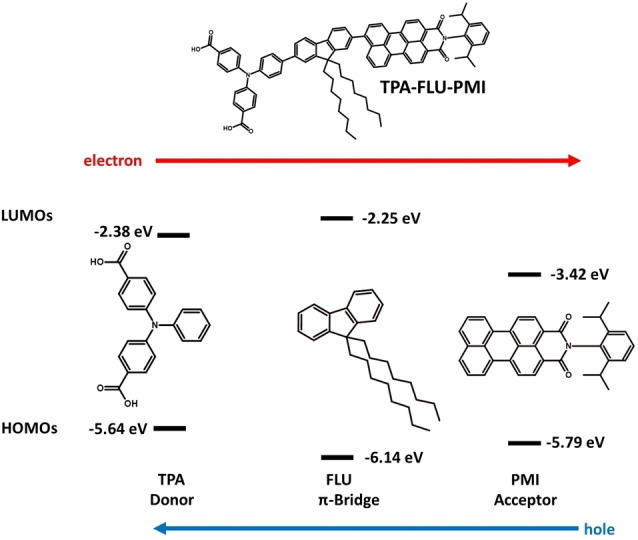
Molecular fragments of the TPA‐FLU‐PMI charge separating dye (chemical structure on top) with orbital energy levels obtained through experimental CV and UV/Vis redox potentials.^[21]^ Due to the higher HOMO energy of the TPA fragment, the hole moves towards the TPA.

The structure of the investigated dyes shown exemplary in Figure 1 is as follows: a PMI‐derived fragment acts as acceptor and photosensitizer. It is connected via a fluorene bridge (FLU) to a polyphenyl amine donor, here, for example, a triphenyl amine (TPA) with carboxylic acid groups. These organic molecules are based on charge‐separating dyes first synthesized and tested by Liu et al.[^9a^, ^9b^] The frontier orbital energies of the involved molecular fragments shown in Figure 1 can be related to the potential measured in cyclic voltammetry (CV) and UV/Vis experiments.^[21]^

Due to the overall decrease in frontier orbital energy from the donor to the acceptor part of the molecule, the excited electron experiences a driving force towards the acceptor, while the hole moves towards the polyphenyl amine donor.

**Scheme 3:**
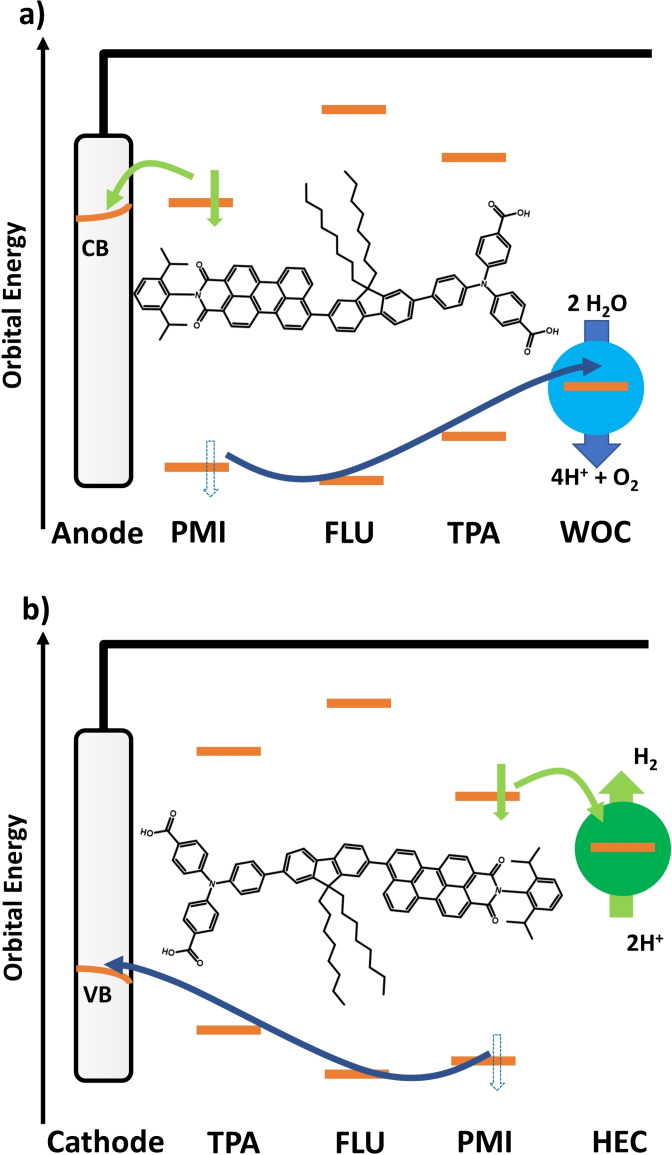
Potential use of a charge‐separating dye in the context of a DS‐PEC. (a) On the photoanode side, where the photoexcited PMI donates the electron (green) into the CB of the anode; the hole (blue) is transferred via the FLU and TPA to a WOC to oxidize water. (b) On the photocathode side, the photoexcited PMI donates the electron (green) to a suitable HEC for hydrogen evolution, while the hole (blue) is transferred via FLU and TPA to be injected into the valence band (VB) of a suitable cathode.

The fluorene acts as both a bridge and small barrier for the hole, while it introduces a large barrier for the electron to prevent electron density moving towards the donor. The electron can then be picked up by an oxidative agent, for example, on the photoanode side by the TiO_2_ electrode, or on the photocathode by a hydrogen evolution catalyst (HEC) as shown in Scheme 3. The hole is either injected from the donor into a semiconductor cathode or used on the photoanode side, for example, to drive a WOC. Also, the attachment of these reaction partners has an influence on the respective frontier orbital energy levels, for example, through deprotonation of the anchoring groups.

Since the donor as well as the acceptor part might already have tuned energetic alignment with those respective reaction partners for further electron and hole transfer, the aim of our study is to find ways to optimize these supramolecular dyes without changing the energy levels of the donor and acceptor. Several different strategies are followed to obtain a better charge separation without changing the driving force significantly. While the fluorene‐PMI based dyes by Liu et al. included a DPA donor,[^9a^, ^9b^] other successful push‐pull systems included TPA donors instead.[^1b^, ^9c^, ^12d^] To estimate which donor would be more efficient in these systems, the effect of a DPA/TPA donor is investigated.

**Scheme 4:**
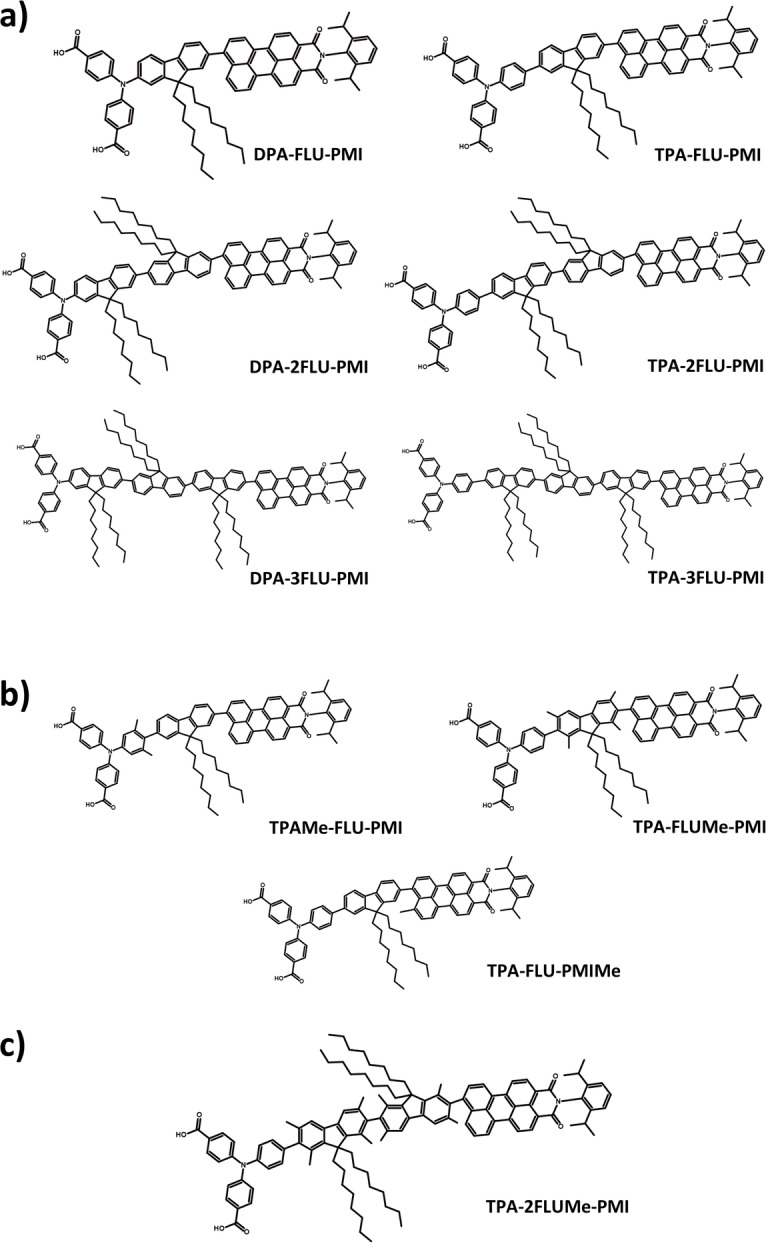
Chemical structures and nomenclature for the investigated dyes with (a) different donor (TPA vs. DPA) and different fluorene chain length. (b) Broken conjugation through introduction of sterically demanding methyl groups. (c) Proposed optimized charge‐separating dye (TPA‐2FLUMe‐PMI): the TPA donor leads to a larger hole density on the donor in comparison to the fluorenes, the chain length of two fluorenes has been shown to give a more efficient charge separation, while the methyl groups on the fluorene molecules break conjugation, lowering the back transfer of hole density to the PMI.

As the fluorene π‐bridge acts as a spatial separation between the donor and acceptor, increasing the bridge length should have a relevant impact on the hole transfer dynamics, as has been shown experimentally in similar molecular systems.[^9b^, ^9c^] Therefore, a further investigation of the effect of the number of fluorene bridge molecules on the charge separation is performed. The investigated supramolecular complexes, including dyes with both DPA and TPA donors and one, two, or three fluorene fragments, are shown in Scheme 4a, including the nomenclature used throughout the paper.

The close coupling between the different components of the charge‐separating dye diminishes the triad character of these supramolecular complexes, with the hole flowing freely between the different components, thus lowering the charge separation efficiency. To decrease the coupling between the components and reduce the back transfer of hole density towards the PMI, methyl groups are here introduced to keep dihedral angles close to 90°. In Scheme 4b, chemical structures of the sterically decoupled molecules are given, with the nomenclature used in the rest of the manuscript.

Our in silico investigation finds that using TPA donors results in a larger spatial separation between electron and hole than DPA donors. Extending the chain length of fluorenes increases the separation efficiency up to two fluorenes, while adding a third fluorene does not significantly improve the charge separation. Introducing methyl groups to decouple the different fragments is most efficient when used on the fluorene bridge. Based on these results we propose an optimized dye that includes the TPA donor connected to the PMI via a bridge that contains two fluorenes, equipped with methyl groups to decouple the fragments. The proposed molecule is shown in Scheme 4c. This molecule shows promise as a more efficient charge separator. The results obtained here are relevant for the optimization of charge transfer processes using push‐pull systems for both photo‐driven water oxidation and hydrogen evolution.

## Results and Discussion

**Figure 2:**
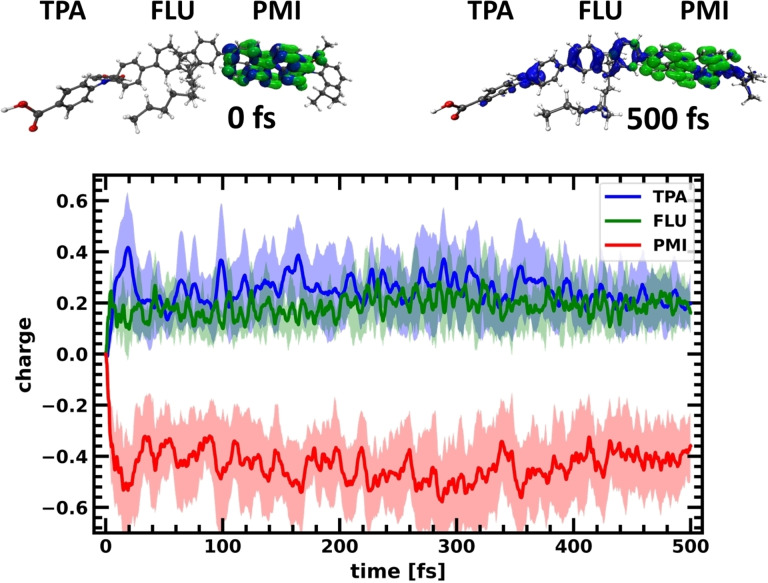
Charge separation in the TPA‐FLU‐PMI dye. Charges are determined as difference between hole and electron population on the respective fragments PMI (red), FLU (green), and TPA (blue). The lines denote mean averages over 10 trajectories, the shaded areas the standard deviation. In the inset above, electron (green) and hole (blue) densities at the beginning and end of one representative quantum dynamics trajectory are shown.

The photoinduced charge separation in the push‐pull system over time is shown for the TPA‐FLU‐PMI case in Figure 2, where the charge corresponds to the hole minus electron population for the different fragments. The lines represent the mean average over the ten charge transfer dynamics (CTD) trajectories, while the shaded area represents the standard deviation.

Upon photoexcitation, both electron and hole reside on the PMI since the excitation is purely excitonic in character (see Tables S5–S14 in the Supporting Information). Thus, the charge difference is initially zero on all fragments. Upon time evolution, the hole migrates to the fluorene and the TPA, while the electron stays at the PMI (see Figure S1 in the Supporting Information), resulting in a negative charge accumulating on the PMI, while the FLU and TPA get positively charged. While the initial charge separation is relatively fast, the charges equilibrate after around 200 fs. In addition, hole transfer is not complete, most likely due to the delocalized character of the HOMO, and the nearly degenerate lower‐lying orbitals (HOMO: −11.47 eV, HOMO‐1: −11.62 eV at optimized geometry, fluctuating during dynamics; see Table S7 in the Supporting Information for the delocalization). In contrast, because of the larger energetic separation for the unoccupied orbitals (LUMO: −9.40 eV, LUMO+1: −8.36 eV), as well as the localized character of the LUMO (Table S7 in the Supporting Information), the electron remains stable on the PMI. After the system reaches equilibrium, the average charge on the PMI corresponds to about −0.44, while the FLU obtains a charge of approximately +0.20 and the TPA of +0.24, based on averaging over the last 200 fs. The charges averaged over the last 200 fs of the three fragments of all investigated molecules can be found in Table 1. The TPA‐FLU‐PMI dye does show photoinduced charge separation, however to an extent that could be further improved.

**Table 1:** Mean charge on the fragments of all investigated dyes, averaged over 10 CTDs and the last 200 fs.

Dye	Donor	Fluorene	PMI	Percentage change^[a]^ [%]
TPA‐FLU‐PMI	+0.24	+0.20	−0.44	0
DPA‐FLU‐PMI	+0.20	+0.26	−0.46	5
TPA‐2FLU‐PMI	+0.21	+0.33	−0.54	23
DPA‐2FLU‐PMI	+0.16	+0.37	−0.53	20
TPA‐3FLU‐PMI	+0.13	+0.41	−0.54	23
DPA‐3FLU‐PMI	+0.13	+0.43	−0.56	27
TPAMe‐FLU‐PMI	+0.28	+0.21	−0.49	11
TPA‐FLUMe‐PMI	+0.26	+0.29	−0.55	25
TPA‐FLU‐PMIMe	+0.22	+0.22	−0.43	−2
TPA‐2FLUMe‐PMI	+0.19	+0.47	−0.66	50

[a] With respect to the charge on the PMI in the TPA‐FLU‐PMI system as reference.

### DPA vs. TPA donor

**Figure 3:**
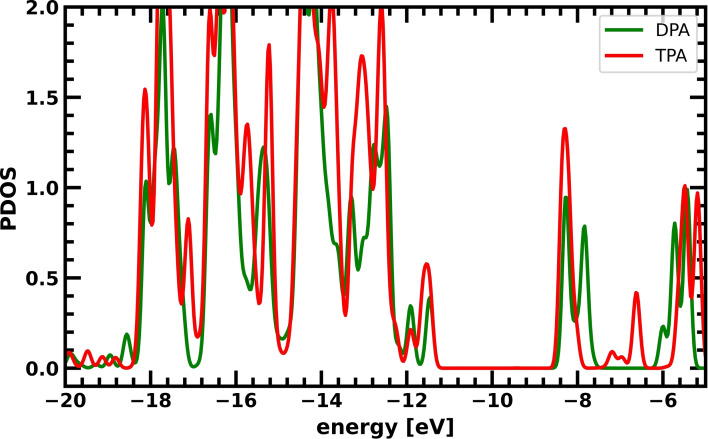
Partial density of states (PDOS) of the TPA (red) and DPA (green) donors.

Introducing a TPA or DPA has little effect on the driving force in these charge separating dyes, as the HOMO energies are similar. This is supported by the density of states of these two fragments (Figure 3). The peaks denoted HOMO (≈−11.6 eV) and LUMO (≈−8.4 eV) in Figure 3 have essentially the same energy for the two systems. Therefore, the boundary condition of a similar donor HOMO energy remains fulfilled even when exchanging DPA and TPA.

**Figure 4:**
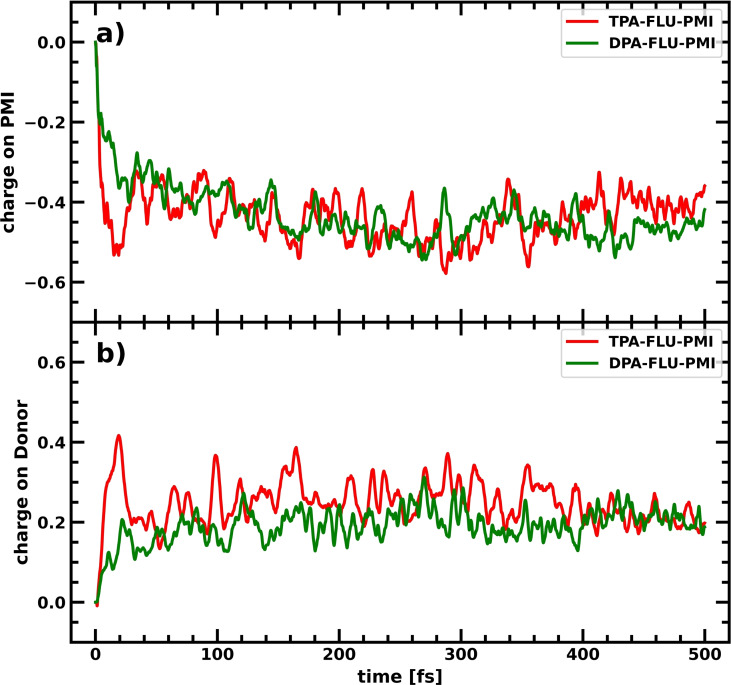
(a) Charge accumulation averaged over 10 CTDs on the PMI in the TPA‐FLU‐PMI (red) and DPA‐FLU‐PMI (green) dyes upon photoexcitation of the PMI. (b) Charge accumulation averaged over 10 CTDs on the donor (TPA/DPA) in the TPA‐FLU‐PMI (red) and DPA‐FLU‐PMI (green) dyes upon photoexcitation of the PMI.

A comparison between the TPA‐FLU‐PMI and DPA‐FLU‐PMI charge separation process in the form of net charge on the PMI after photoexcitation is shown in Figure 4a. Here, the traces represent the charge on the PMI averaged over 10 CTD simulations, while the other fragments as well as the standard deviation are omitted for clarity. The initial charge separation is slightly delayed with the DPA compared to the TPA as donor; however, this difference diminishes after around 100 fs. When averaging over the last 200 fs, the mean charge on the PMI is −0.46 and −0.44 for the DPA‐FLU‐PMI and TPA‐FLU‐PMI dyes, respectively. While the charge on the PMI is not significantly changed, the TPA induces further polarization with a higher positive charge on the donor (+0.24) and lower on the fluorene (+0.20) in comparison to the DPA based dyes (+0.20 and +0.26 for DPA and FLU, respectively) as seen in Figure 4b and Table 1. This holds also true when comparing the DPA‐2FLU‐PMI with the TPA‐2FLU‐PMI molecules (see Figure S2 in the Supporting Information and Table 1).

While the absolute amount of charge transferred seems to be a small amount, it should be considered that the relative increase is the relevant quantity to compare. A theoretically perfect charge separator would have a charge of −1.0 on the acceptor, while the charge on the donor would be +1.0. This perfect charge separator, however, is unlikely to be achieved. In this set‐up of molecules, the close coupling between donor, acceptor, and bridge as well as the finite size of the system results in an incomplete charge separation, but rather a polarization of the molecule. Only when adding an electron or hole sink, as the electrode or catalyst, a complete separation can be achieved. To place our results into perspective, one may keep in mind that the DPA‐FLU‐PMI dye, which here gives a charge of −0.46 on the PMI (meaning about half of the hole has been transferred), has experimentally been shown to be a very efficient charge separator.^[9a]^

In this respect, it is rather remarkable that for such similar systems one may realize with only minor adjustments (not even changing the driving force) a relative increase or decrease of charge on a fragment of tens of percentage points.

For the dyes with TPA donors, the increase of positive charge on the donor in comparison to the DPA‐based dyes (Table 1) corresponds to 20 % (TPA‐FLU‐PMI vs. DPA‐FLU‐PMI), 31 % (TPA‐2FLU‐PMI vs. DPA‐2FLU‐PMI), and 0 % (TPA‐3FLU‐PMI vs. DPA‐3FLU‐PMI).

While a similar amount of hole density is transferred from the PMI, the charge separation is more efficient using a TPA instead of a DPA donor molecule: the TPA leads to larger polarization, as more hole density accumulates on the TPA rather than on the FLU. For an efficient spatial separation, TPA as donor is therefore preferred.

### Increasing the bridge length

**Figure 5:**
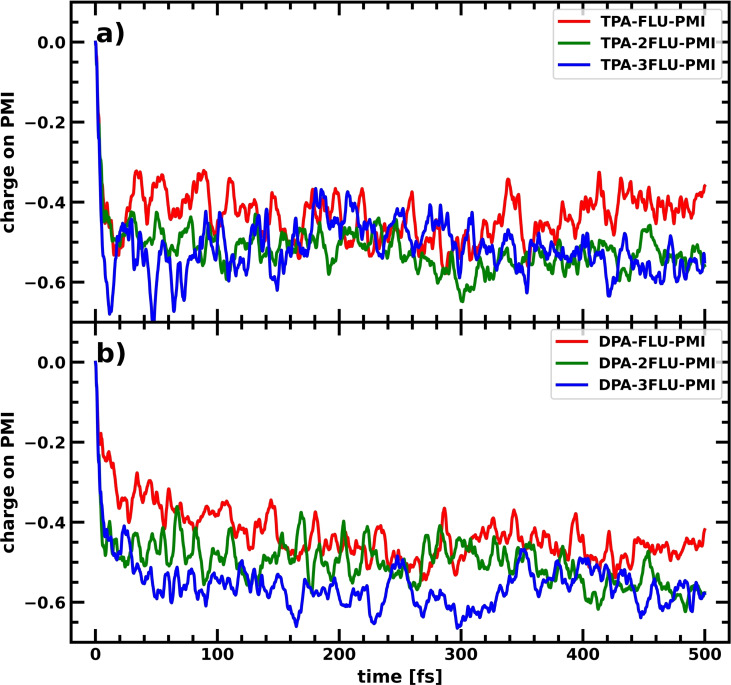
Charge accumulation averaged over 10 CTDs on the PMI with increasing fluorene spacer bridge upon photoexcitation of the PMI. (a) TPA‐FLU‐PMI (red), TPA‐2FLU‐PMI (green), and TPA‐3FLU‐PMI (blue) dyes. (b) DPA‐FLU‐PMI (red), DPA‐2FLU‐PMI (green), and DPA‐3FLU‐PMI (blue) dyes.

In Figure 5, the change in average charge on the PMI fragment of the dyes over time is shown for the TPA‐*X*FLU‐PMI and DPA‐*X*FLU‐PMI molecules, with the number of FLU fragments *X* changing from 1 to 3. Increasing the chain length from one fluorene to two fluorene molecules increases the charge separation significantly for both the TPA‐ and DPA‐based dyes.

Introducing a third fluorene does not further increase the charge separation for the TPA‐based dye, while the DPA‐3FLU‐PMI dye shows an initially better performance than the DPA‐2FLU‐PMI dye that decreases after around 300 fs (see also Table 1). While the negative charge on the PMI increases by about 23 % when extending from the TPA‐FLU‐PMI to the TPA‐2FLU‐PMI, there is no further increase when using the TPA‐3FLU‐PMI dye. For the DPA‐based molecules, increasing the chain length from one fluorene to two fluorenes results in a gain of about 15 % more negative charge on the PMI, with an additional 7 % when adding a third fluorene. In both types of dyes, however, the positive charge on the donor decreases significantly with a 3‐fluorene bridge.

Experimentally, an increase of fluorene chain length has been shown to increase hole injection of the DPA‐*X*FLU‐PMI dyes (differing only in alkyl chain length to our investigated molecules), with the DPA‐2FLU‐PMI dye showing the best performance.^[9b]^ Our computational results also suggest that the charge separation efficiency does not significantly increase when moving from a 2‐fluorene to a 3‐fluorene bridge, since the hole mainly accumulates on the additional fluorenes with limited hole density on the donor (see Table 1). Increasing the bridge length leads to a higher hole delocalization, especially increasing hole density on the fluorenes. This has two interconnected but opposing effects for charge separation efficiency: while the negative charge accumulation on the PMI increases due to the larger hole delocalization, the positive charge accumulation on the donor decreases. In the case of two fluorenes, the negative charge on the PMI increases significantly, while the positive charge on the donor decreases less dramatically. When going from two to three fluorenes, this reduction of positive charge is accompanied by a much lower (DPA‐3FLU‐PMI) or even no increase (TPA‐3FLU‐PMI) of negative charge on the PMI. Here, the increased delocalization along the fluorenes needs to be balanced out to obtain an efficient charge separation.

**Figure 6:**
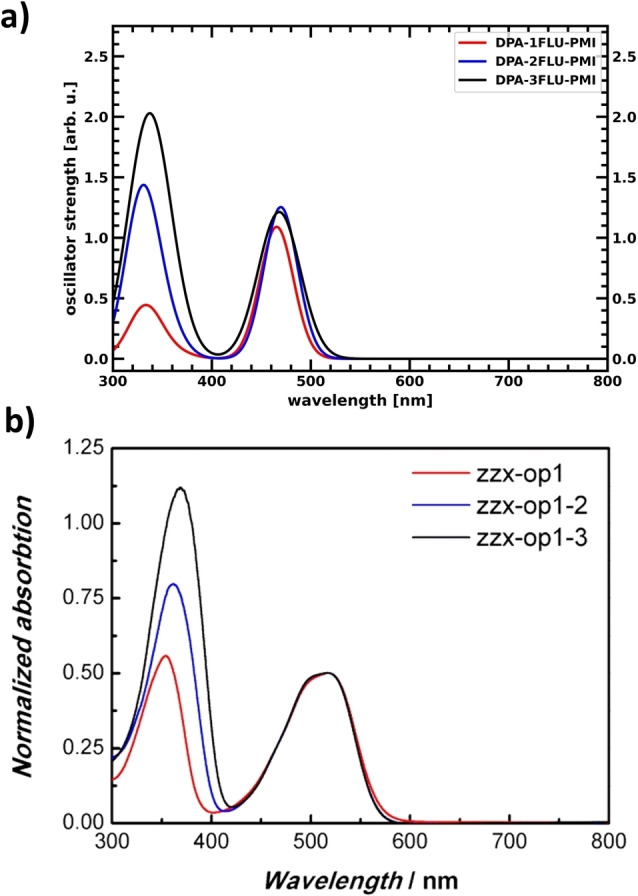
UV/Vis spectra of the charge separating dyes with DPA‐*X*FLU‐PMI structure. Note the increasing oscillator strength/absorption of the lower energetic peak with increasing number of fluorenes. (a) Obtained by Time Dependent Density Functional Theory (TDDFT) calculations (Tables S9, S11, and S13 in the Supporting Information). b) Experimental UV/Vis spectra with zzx‐op1 corresponding to DPA‐1FLU‐PMI, zzx‐op1‐2 corresponding to DPA‐2FLU‐PMI, and zzx‐op1‐3 corresponding to DPA‐3FLU‐PMI. Adapted with permission from: Liu, Z.; Li, W.; Topa, S.; Xu, X.; Zeng, X.; Zhao, Z.; Wang, M.; Chen, W.; Wang, F.; Cheng, Y.‐B.; He, H. Fine Tuning of Fluorene‐Based Dye Structures for High‐Efficiency p‐Type Dye‐Sensitized Solar Cells. *ACS Appl. Mater. Interfaces* **2014**, *6* (13), 10614–10622 (Ref. [9b]). Copyright 2014 American Chemical Society.

Liu et al. attribute the lower efficiency of DPA‐3FLU‐PMI in a photocathode to a lower loading of the molecule on the electrode in comparison to the DPA‐FLU‐PMI and DPA‐2FLU‐PMI molecules.^[9b]^ In addition to this and the lower hole accumulation on the donor, another influence on the decreased performance of the DPA‐3FLU‐PMI and TPA‐3FLU‐PMI molecules in comparison to their counterparts with one or two fluorenes might also be their different absorption behavior. With increasing fluorene length, the fluorene‐based excitonic excitation lowers in energy and increases in oscillator strength, becoming slightly competitive to the PMI‐based excitation as shown in Figure 6, also in agreement with experimental UV/Vis results.^[9b]^ A fluorene‐based excitation requires electron transfer towards not only the PMI but also the donor (TPA or DPA), increasing the likelihood of charge recombination.

In conclusion, increasing the chain length from one to two fluorene molecules leads to improved performance, while the addition of a third fluorene does not lead to significant further improvement. On the contrary, the addition of the third fluorene significantly decreases the hole accumulation on the donor and increases the optical absorption of the fluorenes, thus opening up competing pathways that lead to recombination instead of charge separation, lowering the overall efficiency.

### Decoupling the fragments

**Figure 7:**
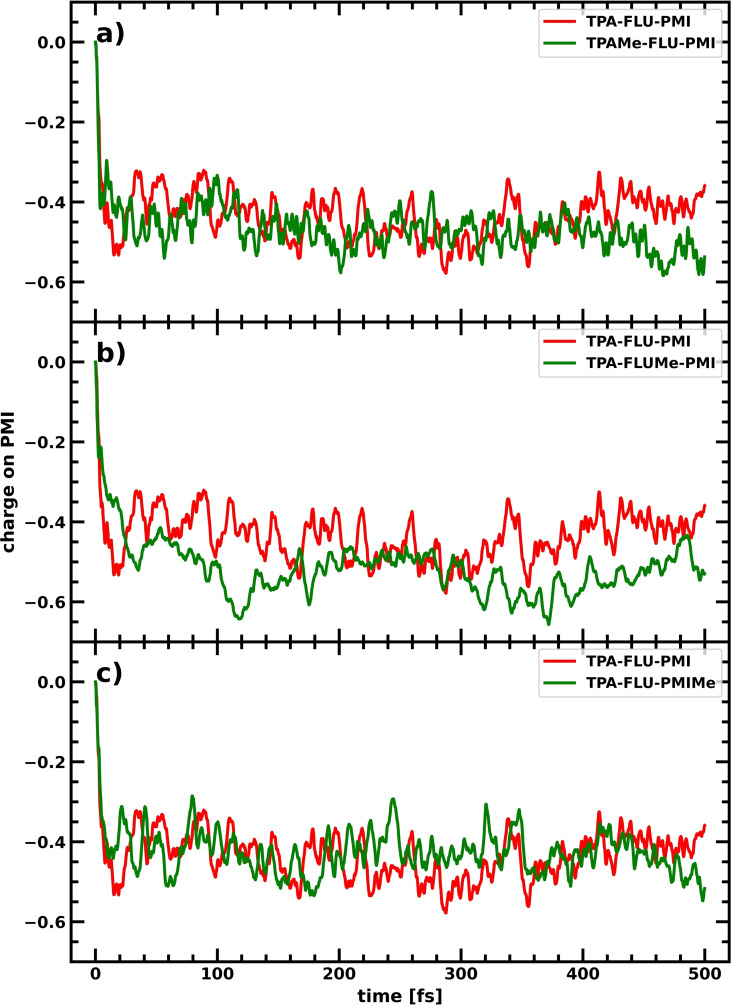
Charge accumulation averaged over 10 CTDs on the PMI in the TPA‐FLU‐PMI case (red) compared to the respective methylated derivatives in green: (a) TPAMe‐FLU‐PMI, (b) TPA‐FLUMe‐PMI, (c) TPA‐FLU‐PMIMe.

One of the problems in the photoinduced charge separation process of these dyes is the relative delocalization of the molecular HOMO, as well as the near degeneracy of some of the highest occupied states, leading to a spreading of the hole over the entire system (see Table S7 in the Supporting Information). One approach to increase the average hole population on the donor would be to limit delocalization of the HOMO by decoupling the different fragments of the molecular dye. This can be achieved by breaking the conjugation between the different components, for example, via forcing the dihedral angle towards a perpendicular orientation and thereby separating the fragments’ molecular orbitals from one another. Photoinduced charge separation should then still be possible, since dynamic fluctuations lead to changes in the dihedrals, allowing charge flow for certain nuclear conformations. While this might slow down initial charge transfer, it should lead to a distinct separation of fragment states, with the HOMO localized on the donor, suppressing back transfer. The inclusion of methyl groups can help enforcing a near perpendicular arrangement of the fragments’ π‐systems by introducing steric strain. Methyl groups were therefore introduced on different parts of the molecule to study the effect on the charge separation process. In Figure 7, charge transfer within the dye is visualized through the net charge over time on the PMI fragment for the original TPA‐FLU‐PMI dye in comparison to dyes equipped with methyl groups at each respective fragment: TPAMe‐FLU‐PMI, TPA‐FLUMe‐PMI and TPA‐FLU‐PMIMe (see Scheme 4b).

While a methyl group on the PMI does not lead to further hole transfer towards fluorene and TPA compared to the reference TPA‐FLU‐PMI molecule, breaking the conjugation through methyl groups on the TPA and FLU leads to a lower net charge on the PMI (see Table 1), thus increasing charge separation in comparison to the planar molecule. Furthermore, the slow‐down of the initial transfer is rather small. Introducing methyl groups to the fluorene seems most promising since more hole density is transferred to TPA and FLU. This is not surprising since methyl groups on the FLU keep both dihedral angles, between TPA and FLU as well as between FLU and PMI, at approximately 90° (see Figure S3 in the Supporting Information). However, larger fluctuations of low frequency are observed than for the coupled case (see Figure 7b). These low‐frequency fluctuations might indicate that the charge separation process becomes much more sensitive to the respective nuclear conformation in comparison to the non‐methylated dyes. Decoupling the fragments from another by breaking conjugation through dihedral angles of roughly 90° can therefore increase the charge separation considerably.

### Optimized charge‐separating dye

**Figure 8:**
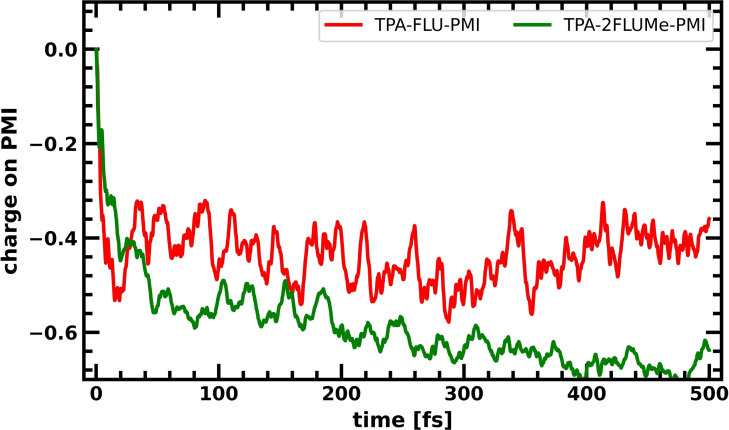
Charge accumulation averaged over 10 CTDs on the PMI for the optimized TPA‐2FLUMe‐PMI (green) dye in comparison to the TPA‐FLU‐PMI (red).

The results presented in the sections above of this systematic in silico investigation can be used to guide the optimization of these fluorene/PMI‐based charge‐separating dyes. As the TPA resulted in more positive charge accumulation on the donor part than the DPA, the TPA was chosen as the donor. Two fluorene molecules were chosen as bridge, as suggested by the results on optimal fluorene chain length. To decouple the different components and suppress the hole back transfer, these two fluorenes were equipped with methyl groups to keep the dihedral angles between all fragments of the molecular system close to 90°, decoupling them and thus keeping their orbitals more localized. All the previous elements are combined in our optimized TPA‐2FLUMe‐PMI dye (Scheme 4c). The resulting net charge evolution on the PMI averaged over 10 CTD trajectories for this system is shown in Figure 8.

The combination of all the modular optimizations of the different components in the supramolecular dye (TPA donor, 2 fluorenes, decoupling through methyl groups) has led to a significantly enhanced charge separation. While the original TPA‐FLU‐PMI dye equilibrates to a charge of about −0.44 for the PMI, in the optimized TPA‐2FLUMe‐PMI dye with the average charge on the PMI at −0.66 (Table 1), equilibrium was not yet reached after 500 fs, with a tendency to make the charge still more negative. Overall, combining the different strategies tested before resulted in a dye optimized in silico that shows an impressive increase of mean charge accumulation by about 50 % relative to the original TPA‐FLU‐PMI.

## Conclusion

Systematic investigations of phenylamine‐Fluorene‐perylene‐monoimide‐based charge‐separating supramolecular complexes were performed at a semi‐empirical quantum classical level to estimate the charge separation in this family of push‐pull dyes. We find that triphenyl amine (TPA) donors lead to a more polarized charge transfer state than for their diphenylamine (DPA) relatives and should therefore be preferred. An optimal bridge length of two fluorene fragments between donor and acceptor has been found to lead to a better charge separation. Expanding the bridge to three fluorene fragments does not significantly improve performance, with potential loss pathways through competing fluorene‐based absorption. Decoupling of the different components in the dyes by sterically demanding methyl groups is an effective way of decreasing back transfer and therefore suppressing the chance of recombination. This is most effective when the methyl groups are introduced on the fluorene. The insights gained from these in silico investigations were then used to propose an optimized charge separating dye using a TPA donor, a bridge of two methylated fluorenes, and a PMI acceptor. This TPA‐2FLUMe‐PMI dye showed remarkable enhancement in charge separation capabilities, with an increase of negative charge by about 50 % on the PMI in comparison to the original TPA‐FLU‐PMI dye. The results gained from this in silico investigation and optimization of charge separating dyes should help in guiding experimental design of these molecules to lower charge recombination, boosting photoelectrochemical device efficiencies, such as the dye‐water oxidation catalyst complexes in the photoanode of a dye‐sensitized photoelectrochemical cell.

## Computational Section

### General procedure

Simulations of photoinduced charge separation in these supramolecular dyes were performed by a combination of the semi‐empirical GFN‐xTB^[16]^ approach to generate a priori classical nuclear trajectories and quantum dynamics simulations of the photoexcited electron and hole using the Atomic Orbital / Molecular Orbital (AO7MO) time propagator and an extended Hückel Hamiltonian.[^17^, ^22^] Density functional theory (DFT) and GFN‐xTB‐based calculations were performed using the Amsterdam Density Functional (ADF) and Density Functional Based Tight‐Binding (DFTB) engines of the AMS2020 program package developed by SCM.^[23]^ The quantum propagation was performed using the program developed by Rego and Batista.^[17a]^

### GFN‐xTB‐based molecular dynamics

The semi‐empirical tight binding approach GFN‐xTB was used to generate the nuclear trajectories. An equilibration run with a total simulation time of 15 ps in the canonical (NVT) ensemble was performed using a Berendsen thermostat^[24]^ at 293.15 K and a time step of 1 fs. This was followed by a molecular dynamics production run in the microcanonical (NVE) ensemble, with a total simulation time of 5 ps, and a time step of 0.1 fs. This trajectory was cut into 10 slices of 500 fs each. Those trajectories were then used in the quantum propagation‐based charge transfer dynamics (CTDs).

### Optimization of extended Hückel parameters

The extended Hückel parameters were optimized to reproduce experimental redox potentials. For this, geometry optimizations of the different molecular fragments were performed using both GFN‐xTB and DFT. For the DFT optimizations, the B3LYP exchange correlation functional with a triple zeta with polarization function (TZP) basis set and D3 dispersion corrections with BJ‐damping, were used.^[25]^ Both GFN‐xTB and DFT lead to very similar geometries, with deviations mostly in the long, flexible alkyl chains. An example is shown for the TPA‐FLU‐PMI dye in Figure S4 in the Supporting Information. The GFN‐xTB geometries were used for the parameter optimization, while the DFT results were used as a reference for the spatial distribution of relevant orbitals. The experimental redox potentials were taken from a manuscript currently in preparation.^[21]^ They were determined via CV and, in cases where the reduction potential was out of measurable bounds, via the main UV/Vis absorption peak onset, which is commonly used to determine reduction potentials.^[26]^ Conversion of these potentials to energies in eV of the frontier orbitals was done by following Equations (1) and (2) for the oxidation potential/HOMO and reduction potential/LUMO, respectively. The target HOMO and LUMO energies $\epsilon_{MOL}^{HOMO}$ and $\epsilon_{MOL}^{LUMO}$ could be estimated from these standard redox potentials versus normal hydrogen electrode (NHE) $E_{ox,NHE}^{0}$ , $E_{red,NHE}^{0}$ with the use of the Faraday constant *F*=*N*_A_ *×e* as given in Equations (1) and 2:

(1) $$\epsilon_{MOL}^{HOMO}\approx -E_{ox,vacuum}^{0}\times nF=-\left(E_{ox,NHE}^{0}+4.44\;\;V\right)\times e$$

(2) $$\epsilon_{MOL}^{LUMO}\approx -E_{red,vacuum}^{0}\times nF=-\left(E_{red,NHE}^{0}+4.44\;\;V\right)\times e$$

Here, $E_{ox,vacuum}^{0}$ and $E_{red,vacuum}^{0}$ are the oxidation and reduction potential versus an electron at rest in vacuum, *n* are the moles of electrons (here one electron, thus 1/*N*_A_), *N_A_* is the Avogadro constant, and *e* is the elemental charge. The resulting energies are then in units of eV. The determined target values can be found in Table S2 in the Supporting Information. The optimization of the extended Hückel parameters was performed on the GFN‐xTB geometries, so that the HOMO/LUMO energies of the respective molecules represent the target values determined in Equations (1) and (2), while the spatial form of the frontier orbitals was checked against the DFT results, to ensure that the obtained orbitals are physically meaningful. The procedure itself is based on a genetic algorithm described elsewhere.^[18a]^ The optimized energies can be found in Table S2, the spatial distribution of the frontier orbitals in Table S6 in the Supporting Information. After optimization of the different fragment molecules on their experimental redox potentials, the TPA‐FLU‐PMI dye gave results remarkably close to the experiment without further optimization (see Table S2 in the Supporting Information). For the methylated molecules, the parameters were not reoptimized as there was no corresponding experimental data available; however, the influence on the energies due to this small structural change is expected to be small.

### Determination of relevant excitations

To characterize the appropriate electron and hole wave packets for the quantum dynamics, relevant excitations in the 400–600 nm wavelength range were determined using linear response (LR)‐TDDFT. The long‐range corrected hybrid functional camy‐B3LYP as implemented in ADF^[27]^ was used for all LR‐TDDFT transitions, with a Double Zeta with Polarization function (DZP) basis, a small frozen core and D3‐dispersion with BJ‐damping. Excitation energies, oscillator strength and transition densities are given in Tables S8–S17 in the Supporting Information. For all investigated molecules, the excitonic PMI HOMO‐LUMO excitation was the most relevant due to its high oscillator strength and excitation energy in the visible light range. These findings were used in preparing the initial hole and electron wavepackets for the quantum propagation.

### Charge transfer dynamics

**Scheme 5:**
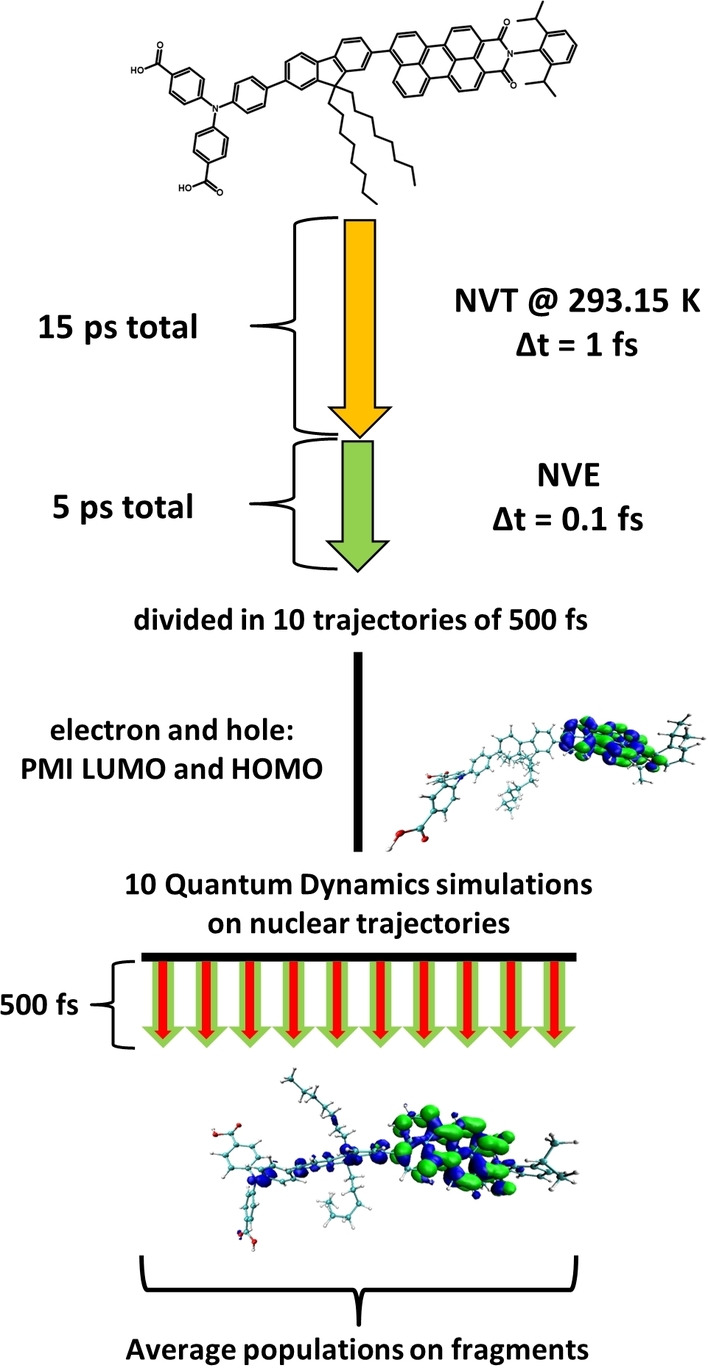
Preparation of the nuclear trajectories and production of the CTDs. A 15 ps NVT equilibration run at 293.15 K is followed by a 5 ps long NVE run with a time step of 0.1 fs. This long trajectory is cut into 10 trajectories of 500 fs each in order to sample different initial conditions for the CTDs. On these nuclear trajectories, quantum propagation of electron and hole, represented by the PMI fragment LUMO and HOMO, respectively, is performed. From these 10 CTD runs, the populations of electron and hole on the respective molecular fragments is averaged to obtain the charge separation within the dye molecule.

Simulations of CTD were performed on the pre‐calculated nuclear trajectories. For each molecule, ten nuclear trajectories of 500 fs length with a time step of 0.1 fs were used, obtained as explained above and shown in Scheme 5. Two wave packets representing photoexcited electron and hole were chosen based on the LR‐TDDFT results: since in all cases, the excitonic PMI excitation is the most intense in the visible region, the wave packets were prepared as the HOMO and LUMO of the PMI fragment. The wave packets were propagated on these a priori nuclear trajectories, with a time step of 0.1 fs for the electronic propagation. After the CTDs, the populations of electron and hole on the different fragments over time were averaged over the 10 trajectories. The changes of charge over time were obtained by subtracting the electron populations from the hole population on each fragment. Average charges were determined over all CTDs and the last 200 fs since at that point the charges were stabilized in most simulations, and in this way quantitative measures of charge separation were obtained. The flowchart of the computational strategy of nuclear trajectory generation and CTDs is given in Scheme 5.

## Conflict of interest

The authors declare no conflict of interest.

1

## Supporting information

As a service to our authors and readers, this journal provides supporting information supplied by the authors. Such materials are peer reviewed and may be re‐organized for online delivery, but are not copy‐edited or typeset. Technical support issues arising from supporting information (other than missing files) should be addressed to the authors.

Supporting Information

## Data Availability

The data that support the findings of this study are available from the corresponding author upon reasonable request.
