# Supplementary material for: In Silico Optimization of Charge Separating Dyes for Solar Energy Conversion
Source: ChemSusChem. 2022 Jun 22;15(15):e202200594. doi: 10.1002/cssc.202200594 (PMC9546488; doi:10.1002/cssc.202200594)
Supplement: Supplementary file 1 — Supporting Information [file CSSC-15-0-s001.pdf]

ChemSusChem

Supporting Information

In Silico Optimization of Charge Separating Dyes for Solar Energy Conversion

Jan Paul Menzel,* Yorrick Boeije, Tijmen M. A. Bakker, Jelena Belić, Joost N. H. Reek, Huub J. M. de Groot, Lucas Visscher, and Francesco Buda*© 2022 The Authors. ChemSusChem published by Wiley-VCH GmbH. This is an open access article under the terms of the Creative Commons Attribution License, which permits use, distribution and reproduction in any medium, provided the original work is properly cited.

SI-1 Electron and hole migration in the TPA-FLU-PMI dye

SI-2 Charge accumulation on the Donor: TPA-2FLU-PMI vs DPA-2FLU-PMI

SI-3 Dihedral angles without and with sterically demanding methyl groups

SI-4 Geometry Comparison between GFN-xTB and B3LYP

SI-5 Optimization of Extended Hückel Parameters

SI-6 Relevant Excitations

SI-1 Electron and hole migration in the TPA-FLU-PMI dye

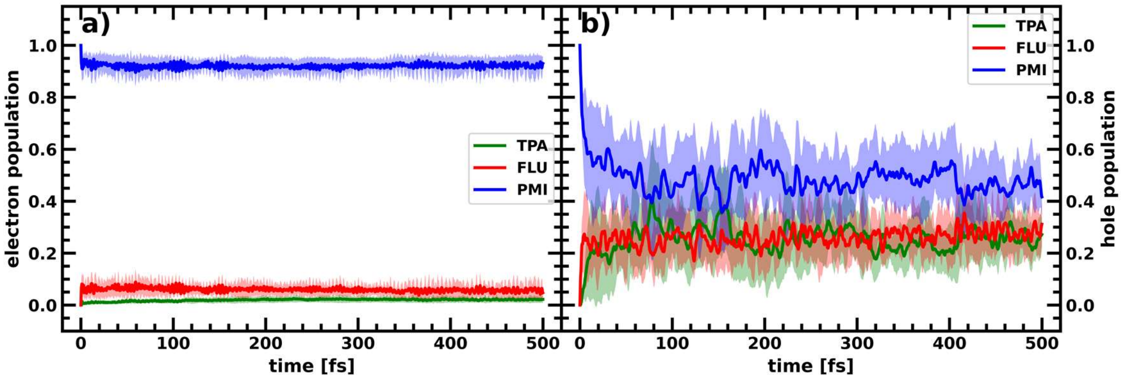

Figure S1. Electron (**a**) and hole (**b**) populations over time on the fragments TPA (green), FLU (red) and PMI (blue) of the TPA-FLU-PMI dye. Only the hole shows significant population transfer.

SI-2 Charge accumulation on the Donor: TPA-2FLU-PMI vs DPA-2FLU-PMI

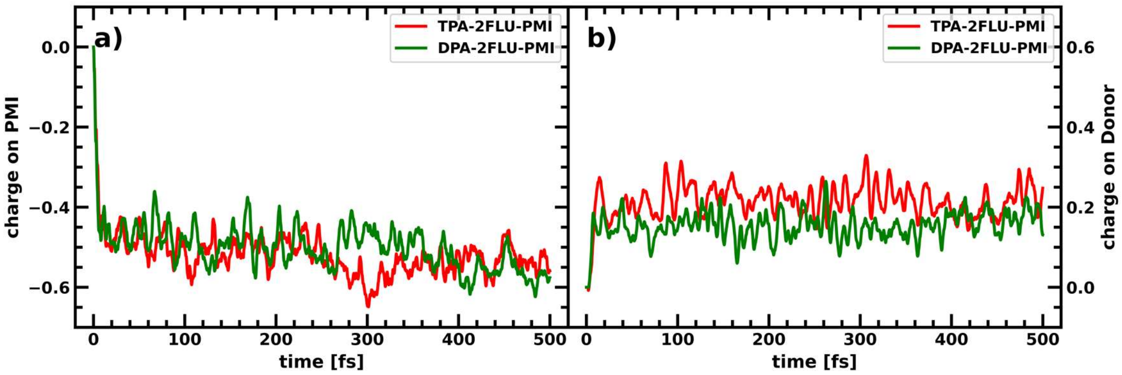

Figure S2. a) Charge accumulation averaged over 10 CTDs on the PMI in the TPA-2FLU-PMI (red) and DPA-2FLU-PMI (green) dyes upon photoexcitation of the PMI. **b)** Charge accumulation averaged over 10 CTDs on the donor (TPA/DPA) in the TPA-2FLU-PMI (red) and DPA-2FLU-PMI (green) dyes upon photoexcitation of the PMI.

SI-3 Dihedral angles without and with sterically demanding methyl groups

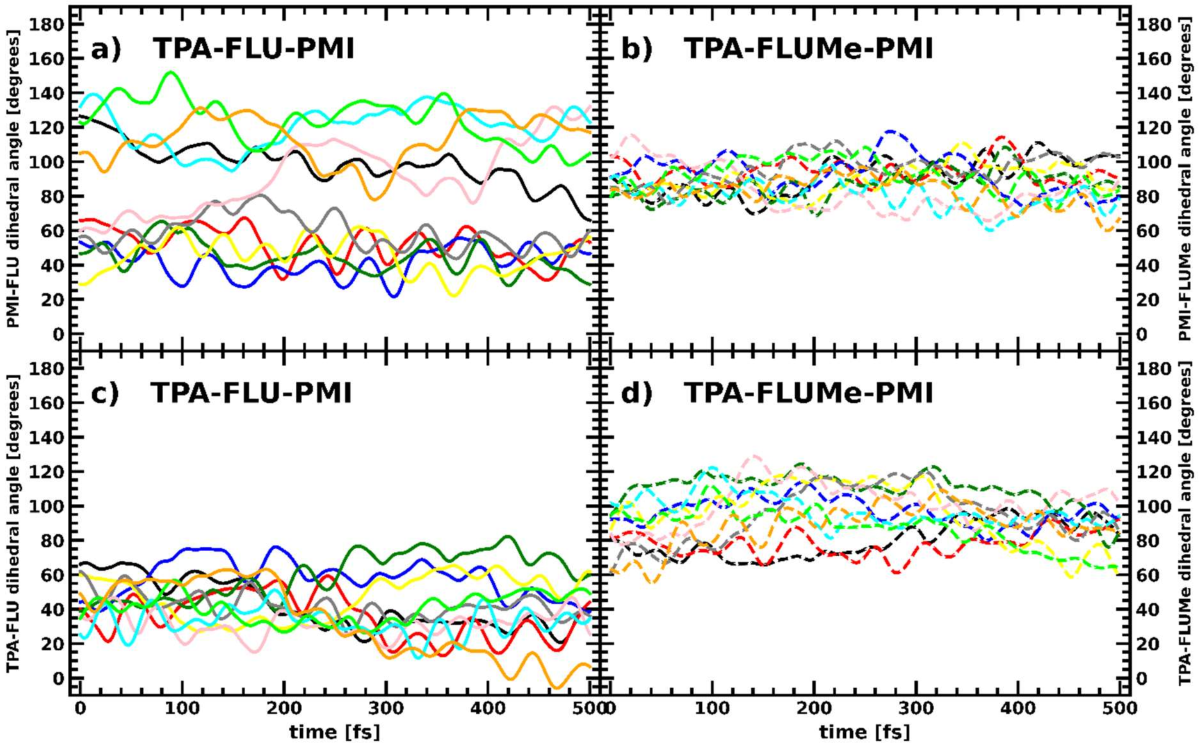

Figure S3. Dihedral angles between the fragments over time for all 10 trajectories **a)** dihedral angle between Fluorene and PMI in the TPA-FLU-PMI dye; **b)** dihedral angle between methylated Fluorene and PMI in the TPA-FLUMe-PMI dye; **c)** dihedral angle between Fluorene and TPA in the TPA-FLU-PMI dye; **d)** dihedral angle between methylated Fluorene and TPA in the TPA-FLUMe-PMI dye.

SI-4 Geometry Comparison between GFN-xTB and B3LYP

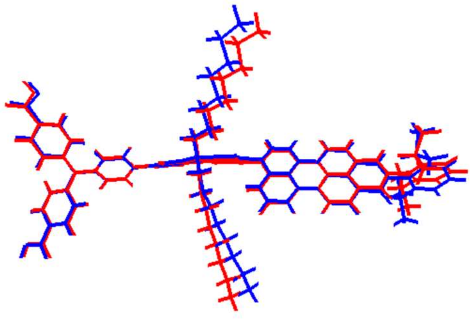

Figure S4. Comparison of the GFN-xTB optimized geometry (red) with the B3LYP optimized geometry (blue) for the TPA-FLU-PMI dye.

SI-5 Optimization of Extended Hückel Parameters

Table S1: *Experimental redox potentials vs. NHE and vacuum from CV measurements, onset of absorption peak in UV-VIS and estimated reduction potential for the three fragment molecules and push-pull dye. Values from reference* [1]

**Molecule**	**CV Oxidation Potential [V]**	**CV Reduction Potential [V]**	**UV-VIS absorption onset [nm]/(eV)**	**Red. Pot. estimate [V] (Ox. Pot - UV-VIS onset)**
TPA	1.20	-	380 nm (3.26 eV)	-2.06
Fluorene	1.70	-	315 nm (3.94 eV)	-2.55
PMI	1.35	-1.02	-	-
TPA-FLU-PMI	1.05	-1.00	-	-

Since the relative alignment of the energy levels are important thermodynamic requirements for the charge transfer, first the PMI was optimized to provide a realistic estimate of the HOMO-LUMO gap. The energies obtained with these optimized parameters (HOMO: -11.80 eV, LUMO: -9.43 eV) had a total shift of 6.01 eV in comparison to the targeted orbital energies, so all target energies were shifted by this 6.01 eV. All other molecules were then optimized to give the same energetic alignment as the experimental values with this 6.01 eV shift. The TPA-FLU-PMI dye was not reoptimized, but the parameters for the molecular fragments were used. The agreement with the target values derived from experiment was very good with deviations in the range 0.02 – 0.05 eV.

Table S2: *HOMO and LUMO energies for TPA, Fluorene, PMI, and TPA-FLU-PMI obtained with standard Hückel parameters, target values generated with a linear shift of -6.01 eV and obtained with the optimized Hückel parameters.*

**Molecule**	**Standard Hückel Parameters [eV]**		**Target values [eV]**		**Optimized Hückel parameters [eV]**	
	HOMO	LUMO	HOMO	LUMO	HOMO	LUMO
TPA	-11.56	-9.45	-11.65	-8.39	-11.64	-8.38
Fluorene	-12.11	-8.89	-12.15	-8.26	-12.15	-8.25
PMI	-11.74	-10.33	-11.80	-9.43	-11.80	-9.42
TPA-FLU-PMI	-11.47	-10.30	-11.50	-9.45	-11.47	-9.40

The parameters and the corresponding atom types used are given in tables S3-5 for the three fragment molecules respectively. The spatial distribution of the frontier orbitals of the fragment molecules determined with B3LYP and the optimized Extended Hückel parameters are given in table S6, while the frontier orbitals of the full TPA-FLU-PMI dye are shown in table S7

Table S3: *Optimized Extended Hückel parameters and atom types used for the PMI fragment*

name	Color in structure	Orbital type	Ionization Potential	ζ	Wolfsberg-Helmholz parameter
**H**	Black	S	-13.60000	1.300000	1.750000
**C1**	Black	S	-21.40000	1.625000	1.750000
		P	-11.40000	1.625000	1.750000
**C2**	Cyan	S	-21.55029	1.603403	1.797230
		P	-11.51291	1.585327	2.171780
**C3**	red	S	-21.62172	1.532642	1.664530
		P	-11.27194	1.702017	1.987490
**C4**	Green	S	-21.42477	1.685850	1.806390
		P	-11.25850	1.657571	1.276690
**N1**	Black	S	-25.93173	1.977443	1.571330
**N1**		P	-12.96157	1.970797	2.215190
**O1**	Black	S	-31.97321	2.202385	1.665370
**O1**		P	-14.51605	1.982010	2.028210

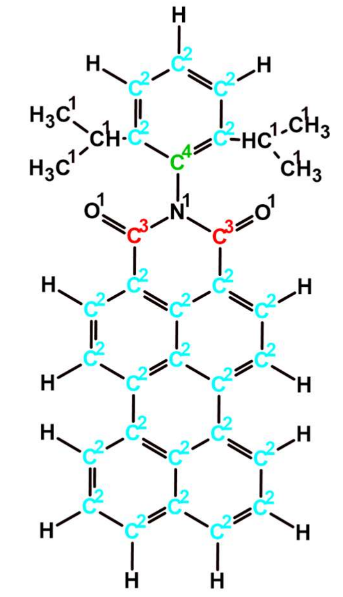

Table S4: *Optimized Extended Hückel parameters and atom types used for the FLU fragment*

name	Color in structure	Orbital type	Ionization Potential	ζ	Wolfsberg-Helmholz parameter
**H**	Black	S	-13.60000	1.300000	1.750000
**C1**	Black	S	-21.40000	1.625000	1.750000
		P	-11.40000	1.625000	1.750000
**C5**	Cyan	S	-21.39014	1.582852	1.525890
		P	-11.30766	1.611940	1.896220
**C6**	Red	S	-21.18695	1.635378	1.619810
		P	-11.41444	1.698127	1.758720

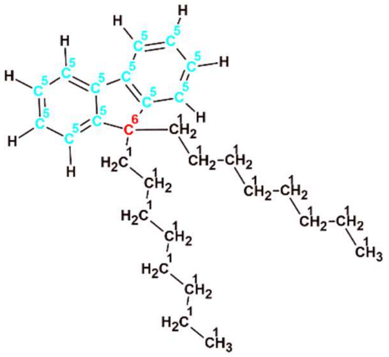

Table S5: *Optimized Extended Hückel parameters and atom types used for the TPA fragment*

name	Color in structure	Orbital type	Ionization Potential	ζ	Wolfsberg-Helmholz parameter
**H**	Black	S	-13.60000	1.300000	1.750000
**C1**	Black	S	-21.40000	1.625000	1.750000
		P	-11.40000	1.625000	1.750000
**C4**	Green	S	-21.42477	1.685850	1.806390
		P	-11.25850	1.657571	1.276690
**C7**	Cyan	S	-21.64227	1.432257	1.631730
		P	-11.16193	1.726305	1.755360
**C8**	red	S	-21.33538	1.548803	1.619300
		P	-11.54327	1.499860	2.361030
**N2**	Black	S	-25.92922	1.971452	1.591160
**N2**		P	-13.10066	2.168376	1.856030
**O2**	Black	S	-31.85792	2.116913	1.948540
**O2**		P	-14.71174	1.975094	2.027960

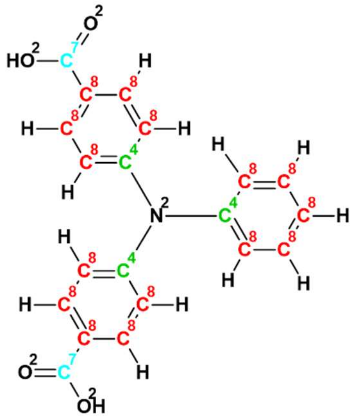

Table S6: *Spatial distribution of HOMO and LUMO for the three fragment molecules, using B3LYP and the Extended Hückel method with optimized parameters.*

	**HOMO**		**LUMO**	
**Molecule/ Method**	B3LYP	Extended Hückel	B3LYP	Extended Hückel
**TPA**	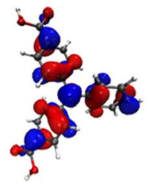	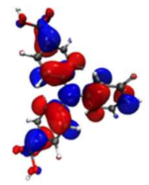	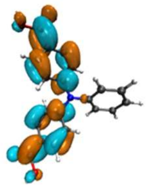	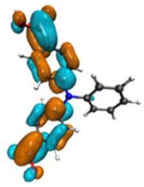
**Fluorene**	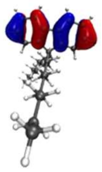	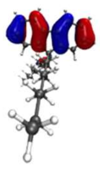	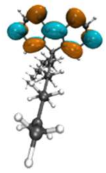	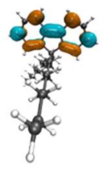
**PMI**	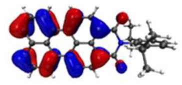	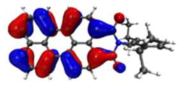	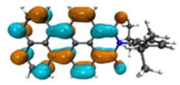	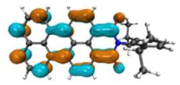

Table S7: *Spatial distribution of HOMO-1, HOMO, LUMO and LUMO+1 for the TPA-FLU-PMI dye, using B3LYP and Extended Hückel method with optimized parameters.*

**Molecule**	**TPA-FLU-PMI**	
**Method**	B3LYP	Extended Hückel
**LUMO+1**	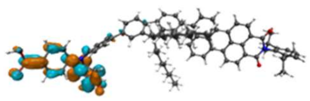	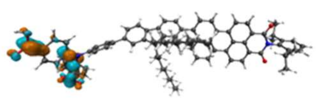
**LUMO**	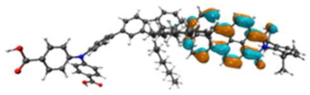	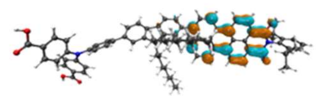
**HOMO**	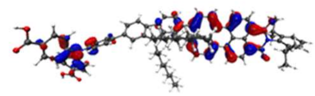	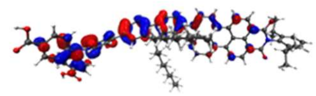
**HOMO-1**	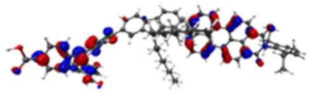	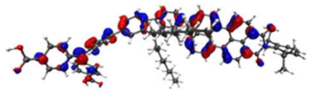

SI-6 Relevant Excitations

Table S8: *TDDFT transitions for the COOH-TPA-FLU-PMI dye, including excitation energy, oscillator strength and transition densities.*

Transition Nr.	Excitation energy [nm]	Oscillator strength	Transition density
1	466 nm	1.117	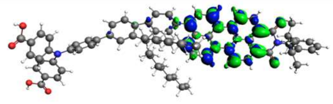
2	352 nm	0.109	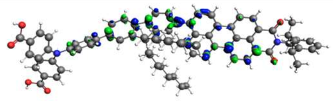
3	330 nm	0.000	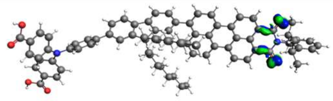
4	329 nm	0.001	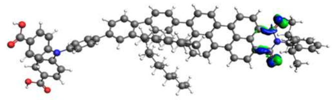
5	327 nm	0.1030	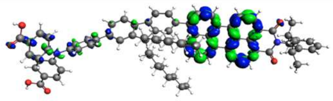

Table S9: *TDDFT transitions for the DPA-FLU-PMI dye, including excitation energy, oscillator strength and transition densities.*

Transition Nr.	Excitation energy [nm]	Oscillator strength	Transition density
1	466 nm	1.090	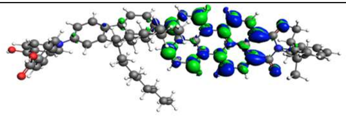
2	365 nm	0.045	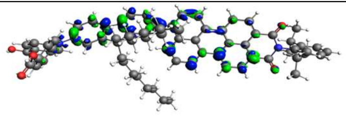
3	333 nm	0.436	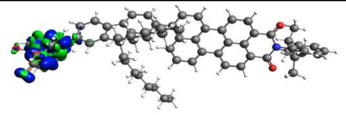
4	330 nm	0.000	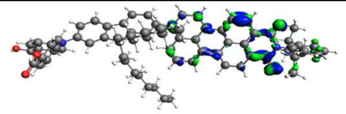
5	330 nm	0.000	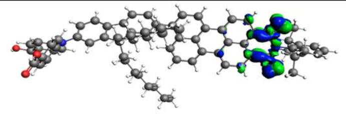

Table S10: *TDDFT transitions for the TPA-2FLU-PMI dye, including excitation energy, oscillator strength and transition densities.*

Transition Nr.	Excitation energy [nm]	Oscillator strength	Transition density
1	470 nm	1.280	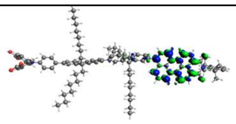
2	359 nm	0.215	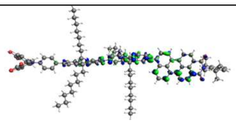
3	332 nm	2.139	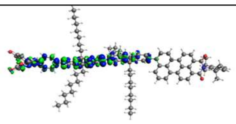
4	330 nm	0.007	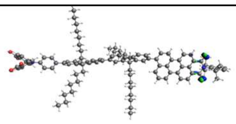
5	329 nm	0.006	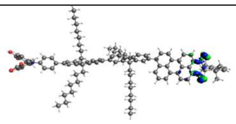

Table S11: *TDDFT transitions for the DPA-2FLU-PMI dye, including excitation energy, oscillator strength and transition densities.*

Transition Nr.	Excitation energy [nm]	Oscillator strength	Transition density
1	470 nm	1.254	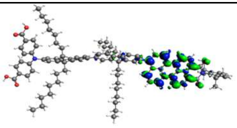
2	356 nm	0.213	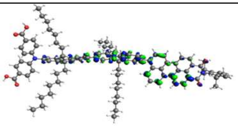
3	331 nm	0.006	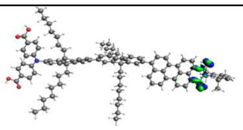
4	330 nm	1.081	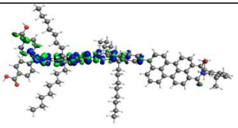
5	329 nm	0.281	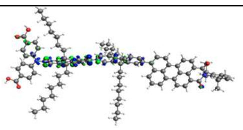

Table S12: *TDDFT transitions for the TPA-3FLU-PMI dye, including excitation energy, oscillator strength and transition densities.*

Transition Nr.	Excitation energy [nm]	Oscillator strength	Transition density
1	469 nm	1.228	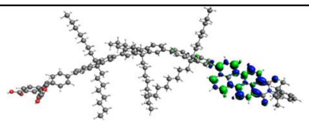
2	356 nm	0.215	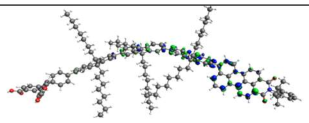
3	335 nm	2.682	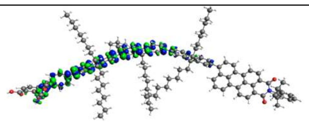
4	330 nm	0.000	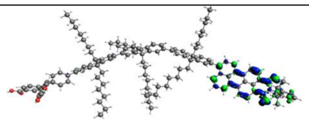
5	329 nm	0.001	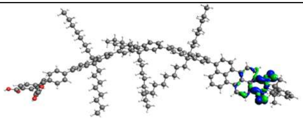

Table S13: *TDDFT transitions for the DPA-3FLU-PMI dye, including excitation energy, oscillator strength and transition densities.*

Transition Nr.	Excitation energy [nm]	Oscillator strength	Transition density
1	468 nm	1.213	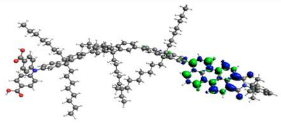
2	354 nm	0.202	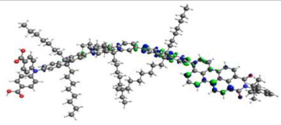
3	336 nm	1.858	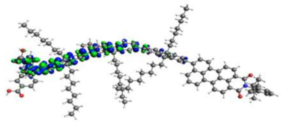
4	330 nm	0.000	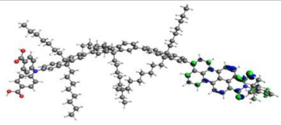
5	327 nm	0.031	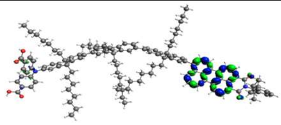

Table S14: *TDDFT transitions for the TPAMe-FLU-PMI dye, including excitation energy, oscillator strength and transition densities.*

Transition Nr.	Excitation energy [nm]	Oscillator strength	Transition density
1	465 nm	1.044	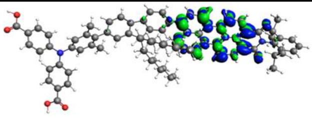
2	337 nm	0.036	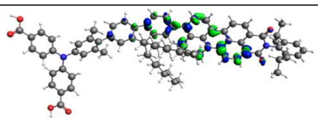
3	330 nm	0.001	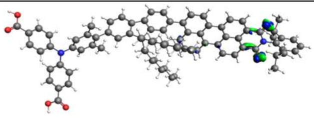
4	330 nm	0.000	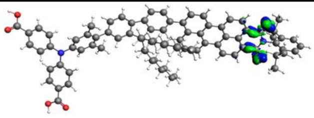
5	324 nm	0.001	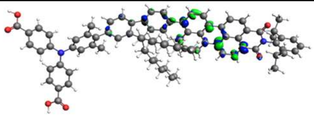

Table S15: *TDDFT transitions for the TPA-FLUMe-PMI dye, including excitation energy, oscillator strength and transition densities.*

Transition Nr.	Excitation energy [nm]	Oscillator strength	Transition density
1	459 nm	0.914	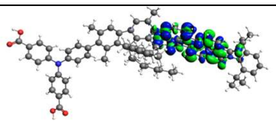
2	365 nm	0.000	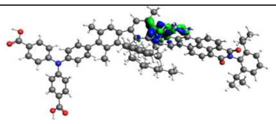
3	328 nm	0.000	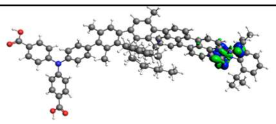
4	328 nm	0.000	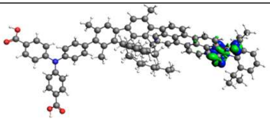
5	313 nm	0.000	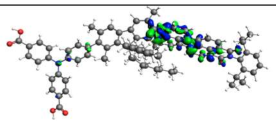

Table S16: *TDDFT transitions for the TPA-FLU-PMIMe dye, including excitation energy, oscillator strength and transition densities.*

Transition Nr.	Excitation energy [nm]	Oscillator strength	Transition density
1	477 nm	1.165	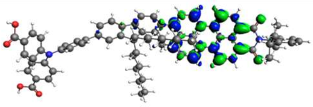
2	353 nm	0.194	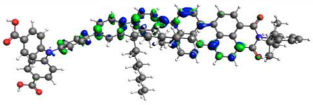
3	330 nm	0.022	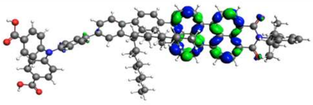
4	329 nm	0.004	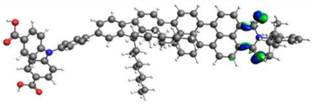
5	328 nm	0.001	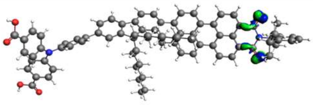

Table S17: *TDDFT transitions for the TPA-2FLUMe-PMI dye, including excitation energy, oscillator strength and transition densities.*

Transition Nr.	Excitation energy [nm]	Oscillator strength	Transition density
1	460 nm	1.038	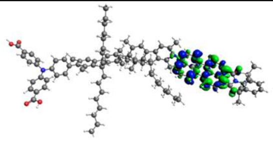
2	369 nm	0.000	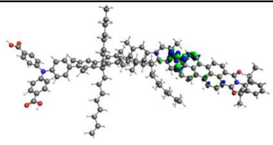
3	329 nm	0.000	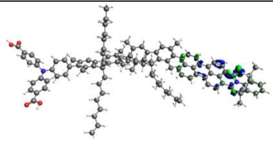
4	328 nm	0.002	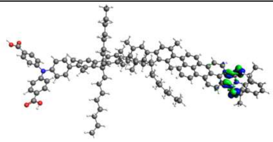
5	327 nm	0.001	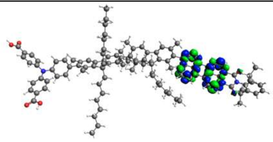

(1) Bakker, T. M. A.; Menzel, J. P.; Vreugdenhil, B.; Bouwens, T.; Buda, F.; Mathew, S.; Reek, J. N. H. Increased Photocurrent by Improving the Donating Properties of the Anchoring Group in P-Type Dye Sensitized Solar Cells. *to be submitted*.
